# The Heart-Placenta Axis in the First Month of Pregnancy: Induction and Prevention of Cardiovascular Birth Defects

**DOI:** 10.1155/2013/320413

**Published:** 2013-04-17

**Authors:** Kersti K. Linask

**Affiliations:** USF Children's Research Institute, CRI #2007, Department of Pediatrics, 140-7th Avenue South, St. Petersburg, FL 33701, USA

## Abstract

Extrapolating from animal studies to human pregnancy, our studies showed that folate (FA) deficiency as well as one-time exposure to environmental factors in the first two to three weeks of human gestation can result in severe congenital heart defects (CHDs). Considering that approximately 49% of pregnancies are unplanned, this period of pregnancy can be considered high-risk for cardiac, as well as for neural, birth defects, as the woman usually is not aware of her pregnancy and may not yet be taking precautionary actions to protect the developing embryo. Using avian and mouse vertebrate models, we demonstrated that FA supplementation prevents CHD induced by alcohol, lithium, or elevation of the metabolite homocysteine, a marker for FA deficiency. All three factors affected the important Wnt signaling pathway by suppressing Wnt-mediated gene expression in the heart fields, resulting in a delay of cardiomyocyte migration, cardiomyogenesis, and CHD. Optimal protection of cardiogenesis was observed to occur with FA supplementation provided upon morning after conception and at higher doses than the presently available in prenatal vitamin supplementation. Our studies demonstrate pathways and cell processes that are involved with protection of one-carbon metabolism during heart development.

## 1. Environmental Influences

Extrapolation of experimental results using mouse and avian embryonic models demonstrates that environmental factors present in utero during the second to third week of human gestation (human, 16 to 19 days after fertilization; mouse, embryonic days ED 6.75 to 7.5; avian, HH stage 4 to stage 5-) can alter early developmental processes resulting in severe cardiac anomalies [1–5]. Some perturbations may alter development in a manner that may not be clinically evident at birth, but result in increased susceptibility to cardiac problems after birth, or increasingly as we age. In a recent study published in September 2012 [6], NIH Common Fund researchers reported on genome-wide association studies (GWASs) of genetic variants, specifically pertaining to noncoding regions of DNA that actively regulate gene expression. They found that 88 percent of GWAS variants are in regulatory DNA regions of genes that are active in *fetal development*, including variants associated with adult-onset disease. The finding that a large number of disease-associated variants are located in regulatory DNA regions that are active during embryonic development suggests that environmental exposures affecting the intrauterine milieu during early embryonic stages can influence risk for not only birth defects, but also a large number of adult diseases. This concept of developmental origins of health and adult disease arose with data that the intrauterine milieu influences cardiovascular and mental diseases long into adulthood [7–9]. 

Early in human gestation following conception, the fertilized embryo implants in the uterine wall. The successful implantation requires adequate maternal uterine perfusion and endogenous hormone preparation to allow initial embryonic survival and later organ development and growth for a term gestation. Shortly after implantation, cardiomyocyte specification and commitment take place between 16–19 days postconception (pc). The circulation and a beating tubular heart are established by 21 days pc in human pregnancy. In mouse development, cardiac specification corresponds to embryonic day (E) 6.75 to E7.5 whereby the cardiac crescent forms. Next, a beating, linear, and tubular heart forms that then loops and septates (E10.5) to form a four-chambered heart (4–6 weeks of human gestation). Human cardiac morphological development is complete by 8 weeks of age.

Abnormal cardiac development leading to congenital heart disease can be associated with abnormal placental development with abnormal trophoblast invasion and remodeling and the resultant abnormal transfer of nutrients and oxygen from the mother to the fetus [10, 11]. Studies relating to germ-line ablation of specific genes, as *p38*α*-mitogen activated protein (MAP) kinase or of peroxisome proliferator-activated receptors (PPARs)*, demonstrated they were critical for proper placental function and, when ablated *only in the placenta*, resulted in cardiac defects. When the placental function was rescued via aggregation with tetraploid embryos (tetraploid morulas failed to contribute to embryonic tissues, only placental), this corrected the cardiac defect [10, 11]. These studies highlight the existence of a critical heart-placental axis that is just beginning to be recognized. 

The heart-placental axis is associated with parallel development of the placenta and heart that utilizes many common molecules and genes [11–16] and reflects intimate and synergistic growth of both organs. Two important pathways critical to placental development, implantation, and cardiac development during the same period of early development are canonical Wnt/*β*-catenin signaling and pathways associated with folate metabolism [17–21]. If these pathways are altered, developmental anomalies ensue. Failure to maintain a pregnancy or have normal cardiac development may be due to abnormal placental function or cardiac function. Fetal loss can occur prior to usual detection of pregnancy at 5 to 6 weeks of gestation (estimated to be a significant percent of all fertilized embryos) or occurs later after initiation of the embryonic heartbeat. Placental volume blood flow is a major determinant of early cardiac output, fetal growth, and well-being [22, 23]. The associated placental abnormality during gestation of the fetus with congenital heart disease has deleterious effects on the outcome of pregnancy. There may be such severe starvation of the fetal circulation and growth failure that growth arrest, severe hypoxemia and marginal cardiac output occur with spontaneous loss of the pregnancy. Premature delivery may result in birth of a neonate with poor prognosis related to the prematurity and low birth weight. Management of the neonate with prematurity and congenital heart disease is associated with a very high perinatal mortality. 

The underlying mechanisms by which environmental exposures can result in congenital birth defects, as well as disease much later in life, have had no clear experimental definitions, but there is evidence that cellular processes associated with cell fate decisions and cell lineage modulation are involved [4, 24]. In the case of cardiomyocyte specification, it is among the earliest cell-fate decisions to occur, because the heart-cardiovascular system is the first organ-system to develop. The statistic that 49% of all pregnancies are unplanned [25], would indicate the first month is a high risk period for the early embryo and specifically for cardiovascular development, neurogenesis, and for placentation in that in all three organ systems important steps in cell specification and early differentiation processes are taking place that are regulated by the same signaling pathways, specifically as we have reported, Wnt/*β*-catenin signaling.

**Figure 1:**
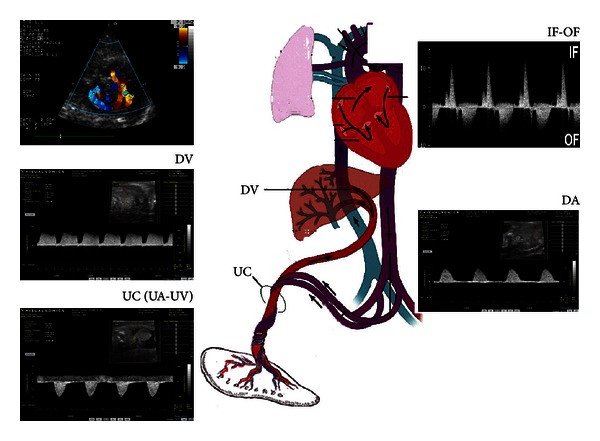
Color Doppler (top left) and pulsed directed Doppler were used to obtain blood waveform patterns. Normal ventricular inflow and outflow (IF-OF) pattern is shown in top right. Locations of waveforms obtained for ductus venosus (DV), descending aorta (DA), and umbilical cord (UC) for umbilical artery (UA) and umbilical vein (UV) are indicated. Permission is granted for the modification of a figure from Merck Source Resource Library, an online Elsevier publication (http://www.mercksource.com).

The focus of my research has been on early stages of heart development, specifically regulation of cardiac cell specification and differentiation using the mouse and avian embryonic models. In the human embryo, a beating, tubular heart with extra- and intraembryonic circulations bringing blood to the heart is present at 21 days. Because cardiac cell fate decisions are taking place several days earlier, approximately at 16 to 19 days of human pregnancy, these early periods of human gestation are not possible to analyze on a cell and molecular level. Thus, the mouse model is an excellent mammalian model to analyze developmental processes leading to a functional heart. With present day technologies, both cardiac structural and functional relationships can be monitored in the mouse embryo using Doppler ultrasound (Figure 1). 

Noninvasive evaluation of fetal heart function during early human pregnancy using clinical echocardiography is challenging. The earliest variables of early fetal cardio-circulatory dynamics have been established for 11–14 weeks of gestation [26]. Those critical developmental processes are already well underway in cardiogenesis by the time pregnancy is usually confirmed at weeks 5/6 after-fertilization which may relate to why congenital heart defects arise in 1% of births world-wide and of all birth defects are the most common. As the heart is the first organ-system to develop, followed closely by the nervous system, our recent research on early development using environmental exposure during gastrulation may help to explain why cardiac birth defects are so prevalent and are the leading cause of infant mortality. In the first month, women may be continuing to use prescribed medications or leisure drugs, to smoke, and to drink alcohol, may be type-2 diabetic or obese, and not be following neither healthy lifestyles or diets, nor protecting their embryos with prescribed perinatal folate supplementation. 

Multiple early processes are associated with similar signaling pathways in heart, placental, and neural development, and thus development of different tissues in the same embryos can be affected with the same exposures [27]. In a recent study of infants in the Atlanta metropolitan area, 28.7% of infants with CHDs also had associated major noncardiac malformations [28]. If one reads the literature provided to women upon learning of their pregnancy usually at 5 to 6 weeks after conception, pamphlets predominantly focus on neural development and the use of folate during pregnancy with targeting the second to third month of gestation. Possibly due to a lack of knowledge on effects of environmental exposures during early embryonic development, generally there is little discussion of adverse effects on cardiac outcomes, importance of planned pregnancy, and the use of folate before becoming pregnant and continuing until pregnancy is confirmed, so as to protect the earliest stages of embryonic development, specifically of the cardiovascular system [5, 18], placentation [29], and neurogenesis [30].

## 2. Cardiac Birth Defects

Our results with animal models demonstrate that even before pregnancy is realized, many different forms of cardiac birth defects can arise with just a single environmental exposure occurring during gastrulation. Cardiac structural and functional defects in mouse embryos we have shown can result with one-time exposure to the drug lithium, alcohol, or homocysteine, the latter a marker of folate deficiency [5, 18]. We analyzed different parameters of the cardiac cycle and umbilical blood flow using ultrasound (Vevo 770, VisualSonics, Inc., Toronto, Ca, instrumentation) with a 40 mHz transducer or a clinical instrument (Philips Sonos 5500, Andover, MA) with a 12 mHz transducer. Both transducers demonstrated similar blood flow patterns. Maternal uterine artery blood flow velocity waveforms were obtained, and the pulsatility index was calculated. Blood flow in the heart and blood vessels was detected in each embryo using two-dimensional real-time echocardiography. Color Doppler was used to identify the embryonic heart and directed pulse Doppler to obtain blood flow velocity waveforms (Figure 1). For venous hemodynamics, we used the presence of flow reversal during atrial contraction (A wave) in the ductus venosus and the presence of umbilical venous pulsations. The abnormal echocardiographic patterns relating to myocardial and valve defects have been published [17, 18]. Our research indicates that the observed cardiac defects are preventable with folate supplementation that is initiated early after conception and at higher doses than presently available in periconceptional vitamins. 

## 3. Common Cardiac Birth Defects Arise with Acute Environmental Exposure to Lithium, Homocysteine, or Alcohol

The causes of congenital heart disease (CHD) can be many, but it is generally assumed that the majority of mutations is not in the fetal organs but in the mother resulting in an altered environment for the developing embryo that predisposes to fetal malformations [32–36]. As changes in cellular processes that relate to cell fate decisions and determination of cell lineages are thought to underlie many birth defects, it is important to have an understanding of the pathways involved during specification and early differentiation stages of the heart-placental axis. What has not been explored are the etiological or simultaneous environmental modulation of *embryonic* cardiac and *placental* functional proteins and developmental pathways. Little research has been done to assay the biomarkers of CHD at a high-risk period of cardiac development, that is, during 3–6 weeks of human gestation, since most women do not know they are pregnant until after the abnormal placental/cardiac microenvironment already has had its deleterious effect on the early stages of the developing embryo. 

**Figure 2:**
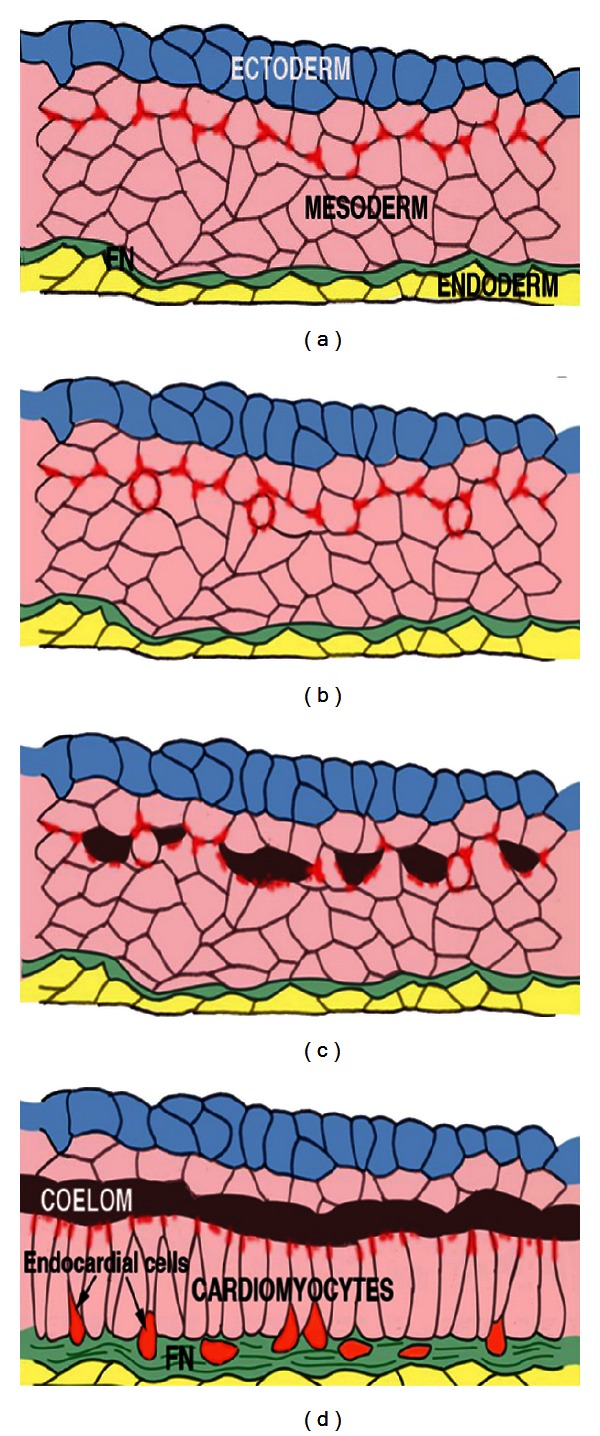
Diagrams depicting the temporal sequence of vertebrate cardiomyocyte differentiation. (a) In the gastrula stage embryo, in the most undifferentiated part of the cardiogenic crescent *β*-catenin localization, an important intermediary of Wnt signaling appears at cell boundaries within the mesoderm. (b) Within this region soon round *β*-catenin-expressing cells are seen. (c) Near these round cells, pericardial coelomata (depicted as black areas) form, as cardiomyocytes change from a mesenchymal organization and elongate to form an epithelial, ventral cardiac compartment. A more ventral population not part of the epithelium now move more ventrally to a fibronectin-rich matrix (green), where these cells (orange) differentiate into endothelial endocardial cells. See Linask review, 2003 [31].

It has been demonstrated that normal heart function relates to formation of normal cardiac structure [37, 38]. Multiple mechanotransducing structures and molecules coordinate detection of blood flow forces with morphogenesis [39–42]. In our mouse studies, we observed that even one-time environmental exposure during gastrulation (ED 6.75 to 8.0; extrapolating to human gestation, 16–19 days postconception) is associated with abnormal umbilical artery blood flow, lower weight fetuses, and both structural and functional cardiac anomalies [4, 5, 18]. Although the mammalian embryo is well protected in the uterus, environmental chemicals, drugs, and maternal nutritional imbalances can interfere with signaling pathways directing placental and embryonic development early in gestation. These environmental factors are thought now to cause at least 7% to 10% of all congenital anomalies [43]. Because biochemical differentiation precedes morphological outcome often by days (Figure 2), the period of susceptibility to environmental chemicals expectedly precedes visible morphogenic effects. 

**Figure 3:**
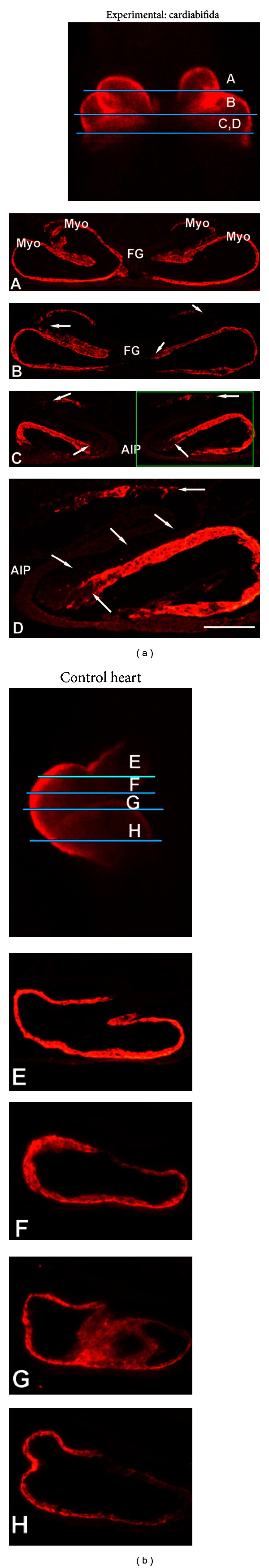
In contrast to the control embryo showing a looping, single tubular heart (right column of figure panels), a lithium-exposed embryo demonstrates a delay in the bilateral heart fields coming together at the midline, and thus a condition of cardiabifida is observed (left column of panels). The experimental, cardiabifida, heart also shows bilateral regions of the heart in which cardiomyocyte differentiation, as defined by MF-20 staining for sarcomeric myosin heavy chain expression, has been suppressed (see areas delineated by white arrows). A–C shows sections through heart from anterior to posterior for the experimental heart. D. A higher magnification of boxed-in region in C is shown here. After the effect of acute environmental exposure dissipates, cardiomyocyte differentiation reappears posteriorly. E–H depict anterior to posterior sections of the control heart. Approximate regions of sections that cut through the heart are shown by lines in the whole mounts of the respective hearts on top.

In our studies, we have focused on embryonic exposure to the drug lithium (Li) widely used for bipolar disorder, the metabolic intermediary homocysteine (HCy) in the folate pathway, or alcohol (ethanol; EtOH). With the same timing of acute exposure, all three induce similar gene misexpression of Wnt-mediated genes *Hex* and *Islet-1* in the cardiogenic crescent during specification. Subsequently, all exposures result in heart, valve, and placental abnormalities [4, 24]. The exposure effects appear to intersect with an early, critical signaling pathway, the canonical Wnt/*β*-catenin pathway, important in the prechordal plate, in cardiac induction, valve development [24, 44–47], and in placentation [48, 49] and neurogenesis [50–52]. Alterations that occur in the placenta and in the heart fields in response to acute exposure of mouse embryos on ED 6.0 at 6 PM, that is, ED 6.75, to Li, alcohol, or elevated homocysteine levels that correlate with changes ~a week later on ED15.5 in umbilical blood flow, abnormal heart (myocardial) function and formation, and valve anomalies in 63.2%, 86.6%, and 66.1% of the embryos, respectively [4, 5, 18]. In comparison to control embryos demonstrating normal cardiac development, there was a high incidence of semilunar valve abnormalities with our timing of the acute exposure. The one-time exposure at E6.75 corresponds to embryonic gastrulation, when the embryo is comprised of only the three germ layers (Figure 2(a)), the ectoderm, mesoderm, and endoderm. This is a time-period during which the primary and second heart fields are being specified (Figures 2(b)–2(d)) in the bilateral anterior mesoderm regions of the embryo [31, 53–57]. Both *Hex* expression, an important inducer of primary heart field specification and *Islet-1*, a marker of the second heart field that leads to the development of the outflow [58, 59], were suppressed with the acute environmental exposure [5, 18]. The delay in regulatory gene expression led to a delay in the migration of cardiac precursors to the embryonic midline resulting in a lethal condition known as cardiabifida and/or to abnormal differentiation of *part* of the heart wall [4]. Dependent on length of exposure and the dose, variability in heart development is seen in regards to degree of cardiabifida and the part of the myocardium that is affected (see Figure 3). Effects of exposure could lead to early embryonic demise, to cardiac anomalies relating to the induction of valves or the conduction system, or to eventual myocardial disease as adults.

Defects in cardiac valves are the most common subtype of cardiac defects, and account for 25%–30% of all cardiovascular malformations [60]. Our published results on lithium exposure administered during gastrulation suggested an additional possible basis for the high incidence of valve defects with early embryonic exposure. We made the observation that early cell populations *extrinsic* to the heart fields arising during gastrulation, specifically the prechordal plate cells [61–64], were susceptible to environmental lithium exposure and failed to contribute in normal numbers to a cell population that localizes to the ventral floor of the foregut and that contributes to the dorsal mesenchymal protrusion (DMP) region [65]. Kirby et al. showed that these cells eventually move into the endocardium [61]. Our lithium exposure studies suggested that this cell population within the DMP arises initially in the prechordal plate, and after migration into the endocardium may become associated with endocardial cushions contributing to valvulogenesis. If the prechordal plate cells are inhibited in their migration as they are by lithium exposure, then cells in the Wnt-expressing valve regions are much more sparse than seen in control embryos and abnormal valvular function was observed in the experimental embryos [4]. Gastrulation is an earlier period of development than generally has been considered to relate to valve abnormalities, although there is evidence from pathological analysis that early cell populations are contributing to valves [65–67]. Valve structural morphogenesis itself takes place much later, that is, after a tubular heart has formed [68, 69].

## 4. Environmental Effects on the Placenta

**Figure 4:**
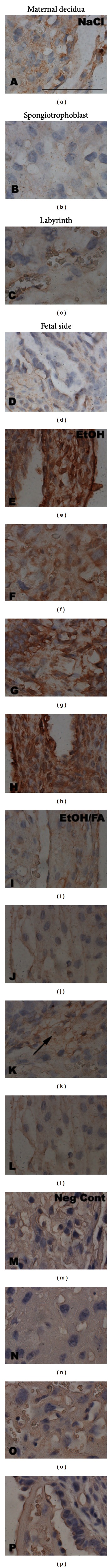
In comparison to placental cells of control embryo ((a)–(d)), one exposure of embryos to a binge-level of alcohol during *gastrulation* resulted in an upregulation of NMHC-IIB in all cell populations of the placenta more than a week after exposure, as observed at mid-gestation (embryonic day 15.5; (e)–(h)). Folate supplementation prevents the upregulation and maintains normal protein expression ((i)–(l)). Negative control is shown in bottom row ((m)–(p)). Figure from Han et al., 2012 [17].

This same time period of embryonic exposure during gastrulation also relates to the formation of the placenta [15, 16, 70]. We recently published that exposure to lithium, homocysteine, or alcohol modulate gene and protein expression in human HTR-8/SVneo extravillous trophoblasts in culture and in vivo in the mouse placentas of the same embryos that demonstrated cardiac anomalies [71]. Central to the placenta's function is its vascular labyrinth, a parallel series of finely branched blood vessels and trophoblasts that regulate nutrient, gas, and waste exchange while keeping the maternal-fetal blood supplies separate. After implantation, angiogenic-vasculogenic processes promote formation of the highly arborized labyrinth vascular bed [72]. In addition to the role of trophoblasts in spiral artery remodeling, migration of trophoblasts in response to chemokines [73] is important in the colonization of the maternal decidua [74] between ED 7.5−10.5 of gestation [75]. The Wnt antagonist Dickkopf-1's modulation of Wnt/*β*-catenin signaling plays a critical role in regulating the equilibrium between active and inactive Wnt signaling in the utero-placental interaction [76, 77]. Although human placentation differs from that of the rodent model, there is homology regarding trophoblast invasion and in the important regulatory elements [78]. We found that alcohol exposure as well as lithium and homocysteine did not affect cell proliferation, but rather trophoblast cell migration [17]. All three environmental exposures modulated placental nonmuscle-myosin-heavy-chain-(NMHC-) IIA and NMHC-IIB expression (Figure 4). NMHC-IIA has been reported to have a unique role in the placenta and when deleted embryonic lethality ensued [79]. NMHC-IIB, a protein associated with cell motility, also has a role in heart looping and trabeculation [39, 80, 81]. 

## 5. Folate Protection of Normal Heart-Placental Axis Formation and Prevention of Birth Defects

There is a general acceptance that folate aids in the prevention of neural tube defects. There is an increasing evidence, including our own, that folate supplementation can prevent or reduce the risk and severity of congenital heart disease induced by an abnormal uterine microenvironment. Prenatal treatment with folate has been shown to prevent neural tube defects and reduce the severity of CHD in clinical studies [82–88]. In animal analyses using chick and mouse models, supplementation of folate or simultaneous supplementation of folate and myo-inositol have been shown to prevent the teratogenic effects of a number of environmental molecules that can affect human gestation, including alcohol [5, 18]. In human epidemiological studies, folate doses of 10 mg/kg have proven effective in preventing cardiovascular defects [87]. In our mouse studies, a metabolic weight dose equivalent of 10.5 mg/kg maternal weight completely prevented cardiac defects induced during gastrulation [5, 18], but a more moderate dose of 6.2 mg/kg provided only partial protection. No deleterious effects on normal development were noted with these doses. These results suggest that a slightly higher dose of folate may be necessary for the prevention of cardiac birth defects than for neural tube defects where 1 mg/kg FA may be given for high risk pregnancies. A mouse study using a folate dose 4 times higher than ours, that is, 40 mg/kg, showed that such a high dose was deleterious to normal embryonic development [89]. It appears that these investigators were using a dose within a toxic range. It is suggested that more clinical studies are needed to define an optimal FA dose for the prevention of CHD in human pregnancy. 

The folate pathway not only relates to purine and pyrimidine synthesis important in DNA synthesis and cell proliferation, but also to the synthesis of the primary methyl donor S-adenosylmethionine (SAM) important in methylation reactions of cellular lipids, proteins, RNA, and DNA. DNA methylation is critical to epigenetic regulation of gene expression [90–92]. Epigenetic factors that predispose to congenital heart disease, and placental dysfunction are suspected to be the cause of an increase in the recurrence risk of CHD after one affected child. During cell differentiation, gene expression patterns are controlled through two principle mechanisms. The best understood is imposed through the DNA sequence itself. The second or “epigenetic” relates to heritable changes in gene function that occur independently of alterations in primary DNA sequence. The best-characterized epigenetic modifications are DNA methylation and histone modifications, both of which function in Wnt signaling, a critical pathway in early cardiomyogenesis [24, 44, 45, 47, 93], neurogenesis [50, 94–96], and placentation [48]. Vertebrate DNA methylation is in general restricted to cytosine (C) nucleotides in the sequence CG, known as CpG islands. DNA methyltransferases (Dnmts) are the enzymes responsible for DNA methylation. A principle source of methyl groups in the cell is S-adenosyl methionine (SAM) synthesized by the FA metabolic cycle. In the early embryo, DNA after fertilization undergoes progressive demethylation and becomes hypomethylated during the pluripotential stages [97]. As cells differentiate, a more hypermethylated state is attained, as cells “lock-in” their own specific fate and other genes are silenced through methylation [98]. Thus, specificity of DNA methylation patterns is expected for specific cell types. However, recent evidence indicates that neither all genes are methylated with differentiation nor all genes that are methylated are necessarily silenced. Epigenetics are important areas for analysis in relation to birth defects.

Canonical Wnt signaling is necessary for the induction of cardiogenesis [44] to occur, but if potentiated or prolonged beyond when it is normally downregulated in the heart fields by Wnt antagonists such as Dkk-1 and by Wnt 11, it becomes inhibitory to differentiation [24, 47, 99]. In the human *DKK-1* gene expression involves regulation by DNA methylation within its promoter [100]. Recent reports demonstrated that alcohol exposure altered DNA methylation profiles in mouse embryos at early neurulation [101] and the patterning of 5-methylcytosine expression during neurogenesis [102]. Another study reported on a correlation between chronic alcohol use, for example, and demethylation of normally hypermethylated imprinted regions in sperm DNA [103]. Thus, as based on our studies and that of others, analyses of one-carbon metabolism and specific epigenetic effects of embryonic environmental exposure in the induction of placental and cardiac anomalies and of folate in the prevention of abnormal development are warranted. Given that a high percentage of pregnancies are unintended, the mechanism of action of the folate and intersecting pathways during early gestation together with prophylactic mechanisms involving epigenetic effects are critical to define in the placenta-heart axis. 

There is an increasing awareness in the last 4 to 5 years that increased levels of attention are necessary to be brought to environmental exposures occurring in the first month of pregnancy in the etiology of congenital heart defects [104]. Epidemiological studies and statistics provide the basis for the necessity to study early embryonic development in relation to the uterine environment and the formation of birth defects. 120,000 to 160,000 children are born with major birth defects each year (3%–5% of all live births). Twelve percent of admissions to pediatric hospitals relate to children with birth defects [105]. Not withstanding the stress and toll of birth defects to the child and family, fifteen of the most significant birth defects have a cost to society of $8 billion annually. Genetic mutations and chromosomal abnormalities account for approximately 28% of birth defects among affected individuals [106]. More than 66% of birth defects, however, relate to unknown, most likely multifactorial causes, including environmental influences. Understanding how and when in utero environmental exposures affect embryonic gene regulation and which pathways are perturbed will provide insights into ways to prevent disease and birth defects by reducing effects of exposures early in life, instead of treating the anomaly often by surgery or the disease when symptoms occur in adulthood.
